# Influence of provenance origin on the early performance of two sclerophyllous Mediterranean species established in burned drylands

**DOI:** 10.1038/s41598-021-85599-3

**Published:** 2021-03-18

**Authors:** Sergio E. Espinoza, Marco A. Yañez, Eduardo E. Martínez, Marcos R. Carrasco-Benavides, Suraj A. Vaswani, John Gajardo, Carlos R. Magni

**Affiliations:** 1 Departamento de Ciencias Forestales, Facultad de Ciencias Agrarias y Forestales, Universidad Católica del Maule, Avenida San Miguel 3605, Talca, Chile; 2 Núcleo Científico Multidisciplinario, Universidad de Talca, 2 Norte 685, P.O. Box 747, Talca, Chile; 3 CESAF, Facultad de Ciencias Forestales y de la Conservación de la Naturaleza, Universidad de Chile, Avenida Santa Rosa 11365, La Pintana, Chile; 4 Departamento de Ciencias Agronómicas, Facultad de Ciencias Agrarias y Forestales, Universidad Católica del Maule, Los Niches-Curicó, Chile; 5 Facultad de Ciencias Forestales y Recursos Naturales, Instituto de Bosques y Sociedad, Universidad Austral de Chile, Campus Isla Teja, Valdivia, Chile

**Keywords:** Plant sciences, Plant physiology

## Abstract

Forest restoration have had limited success due to intense and prolonged droughts in Mediterranean-type ecosystems. In this context, knowledge of growth and physiology in seedlings of different provenances can be useful in the selection of appropriate seed sources for restoration. In this study we investigated variations in survival, growth, and leaf-level physiology of five provenances of *Quillaja saponaria* Mol. and five provenances of *Cryptocarya alba* Mol. originated from coastal and Pre Andean sites exhibiting latitudinal-related climate differences in central Chile. Seedlings were grown in a nursery on 600 mL pots for 18 months and then planted in a dryland site severely damaged by fire. One year after establishment, we measured survival, growth, and leaf-level physiology. We also analyzed the relationship between outplanting survival with seedling characteristics prior to planting, and the relationship between growth and survival with physiological traits and with climate variables. Growth and survival were similar among provenances of *Q*. *saponaria* and *C. alba*, with the exception of differing heights observed within the provenance of *Q*. *saponaria*. Initial root collar diameter of *Q*. *saponaria* was observed to be positively correlated to outplanting survival. With the exception of photosynthesis in *Q*. *saponaria*, all provenances of both species differed in the leaf-level physiological traits. Those provenances originating from interior dryland sites exhibited lower stomatal conductance and used water more efficiently. The opposite was true for provenances coming from coastal sites. In outplanting sites with Mediterranean-type climates that have been damage by severe fire, selections based on larger diameter seedlings, especially for *Q*. *saponaria* and from interior and pre-Andean provenances, will likely improve outplanting success.

## Introduction

Mediterranean-type forest ecosystems are very fragile and susceptible to degradation^1^. In South America, the Chilean Mediterranean-type forest ecosystem has been under permanent threat since the European colonization and 83% of its original coverage is already lost mainly because of threats such as land conversion for agricultural purposes^2^. The restoration of this degraded ecosystem have had limited success and have created concern about the factors influencing the success for its restoration, which may greatly depend on factors such as the appropriate seed sources^3^ and seedling morphological attributes determining the quality of the planting stock, i.e., desirable phenotypic traits; such as shoot height, stem diameter and root system, that promotes successful seedling establishment^4,5^.

Provenance origin and seed source plays an important role in the successful restoration of degraded areas^6^. Local seed sources are better adapted to local conditions, which improves survival and growth^7^ as has been found in *Quercus ilex* L. and *Quillaja saponaria* Mol.^8,9^. Adaptations of sclerophyllus species growing across a range of Mediterranean-type climates has shown that provenances from mesic environments have elongated and large leaves^10^, while provenances from dry climates have a reduced leaf area as an adaptation to face water restriction^11^. Stomatal regulation in response to drought stress is an important mechanism of Mediterranean sclerophyllus species such as *Q. ilex* to prevent water loss^12^. The species exhibits early stomatal closure before to postpone the onset of water stress^13^.

Seedling quality is another important factor that constrains the success of the restoration because it influences plant survival and growth^14–17^. Seedling quality is assessed by several morphological and physiological attributes, but height, diameter, and root:shoot ratio are typically considered as good predictors of the outplanting survival^5^. In general, large seedlings increases their survival and growth in degraded drylands because they develop larger roots that can reach moisture in deep soil horizons^16,18–23^.

*Quillaja saponaria* Mol. and *Cryptocarya alba* Mol. are two endemic sclerophyllus tree species widely distributed in the Mediterranean drylands of central Chile. Populations of both species were severely degraded by the fires occurred in central Chile during summer 2017, being the worst catastrophic wildfires of the last 50 years that burned nearly 520,000 ha of land^24^. This situation aggravated the conditions for forest recovery, and exacerbated the early mortality of planted seedlings^9,25^. The distribution range of both species spans from the coastal areas in the arid northern part of Chile (30° S) to the humid climates in the southern part of the country (40° S)^26^. Both species have specific adaptations to survive in Mediterranean-type climates. *Q*. *saponaria* is a shade-intolerant and deep-rooted species that exhibits water potentials of c.a. − 2.0 MPa during summer, while *C*. *alba* is a shade-tolerant and shallow-rooted species that maintain water potentials of c.a. − 4.0 MPa in the summer period^9,27,28^. *Q*. *saponaria* has a higher photosynthetic rate and stomatal conductance than *C*. *alba*^29,30^. Although both species are routinely planted in restoration projects, its outplanting performance is highly unpredictable and variable^25^. There is still a gap in the information on the outplanting performance after a severe fire in relation to seed-source provenance for both species, causing concerns of the appropriate seed sources for restoration under climatic change context. Severe fires are known to cause losses of organic matter and nutrients, increases soil erosion^31^ and spatial variation of the soil properties, decreases in site productivity^32^, and plant growth, and survival^33^, and decreases in post-fire recruitment^34^. As seedlings experience higher rates of mortality in the first year after planting, monitoring the early performance can give guidelines for achieving restoration goals. Thus, the aim of this study was to assess variation in growth, survival, and leaf-level physiology across a latitudinal gradient of seed sources for *Q*. *saponaria* and *C*. *alba* that were established on a dryland site severely damaged by fire. We hypothesized that provenance origin will have a great effect on variation in outplanting success of *Q*. *saponaria* and *C*. *alba* in harsh sites.

## Methods

### Study site

The experiment was established at Las Brisas Experimental Station (35° 34′ S, 72° 06′ W, 254 m a.s.l) which belongs to the Universidad de Chile and it is located 50 km west of the San Javier city, Maule Region, central Chile. In 2017, the total area of the experimental station (93 ha) was completely burned by fire (de la Barrera et al. 2018). We estimated the burn severity at the study site, based on the relativized delta normalized burn ratio (RdNBR) from pre- and post- fire Sentinel-2 (20 m) images. The RdNBR is an index developed from satellite imagery to estimate vegetation burn severity maps using pre and post-fire images. The index values usually can be grouped into three categories as low, medium, and high severity, depending on alterations in the soil, canopy cover, and vegetation mortality^35,36^. The results indicated that a high percentage of the study area experienced a high burn severity. Prior to the fire, the area supported different vegetation types such as: (1) isolated trees of native forests, dominated by species such as *Q. saponaria*, *Acacia caven* (Mol). Mol., *Lithraea caustica* (Mol.) Hook. & Arn., *Escallonia pulverulenta* (Ruiz et Pav.) Pers., and *Peumus boldus* Mol., (2) plantations of *Pinus radiata* D. Don, and (3) introduced pasture grasses such as *Aira cariophyla*, *Briza minor*, and *Bromus hordeaceus* among others. The climate at the study site is considered Mediterranean, with annual rainfall of 734 mm occurring mostly in the winter months (675 mm from June to August). The dry period is around 7 months, and summer is typically hot and dry (maximum daily temperature of 36 °C during January). The soil is neutral (pH 6.1), sandy clay (47% sand, 17% lime, 36% clay), low electrical conductivity (0.03 dS m^−1^), and 1.5% organic matter content. The available soil nutrient content is 4 mg kg^−1^ N, 8 mg kg^−1^ P, and 168 mg kg^−1^ K. Based on SPAW Hydrology (a free online water budgeting tool from the USDA Natural Resource Conservation Service) we obtained volumetric water content and bulk density at a depth of 30 cm. Values were 8.42 cm at field capacity, 5.65 cm at wilting point, and 1.49 g cm^−3^ bulk density.

### Plant material

**Figure 1:**
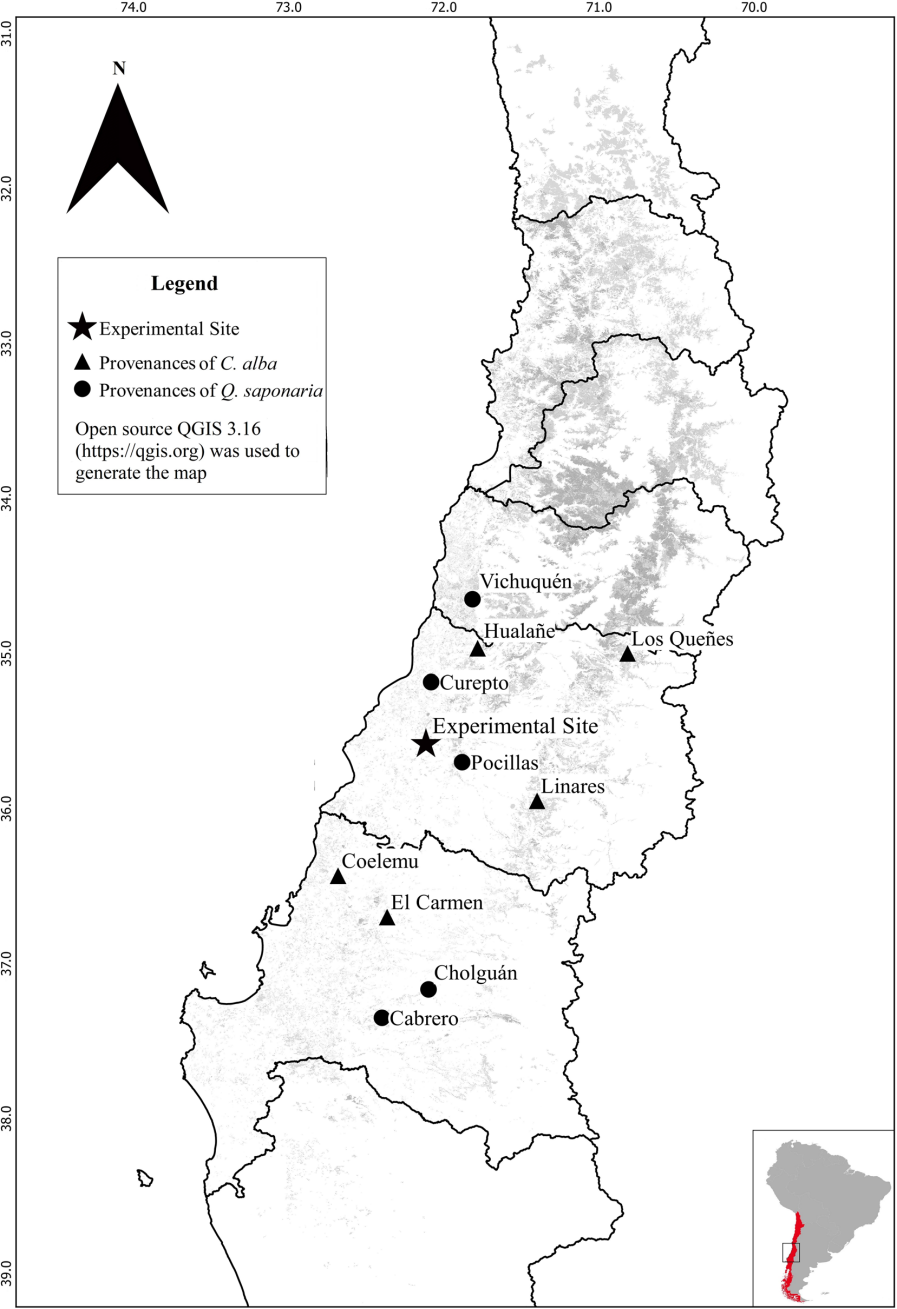
Location of the provenances origin and the experimental site. The approximate range of distribution of both species in central Chile is indicated by a square in the inset graph.

Five provenances of *Q*. *saponaria* and *C*. *alba* exhibiting latitudinal-related climate differences (Table 1, Fig. 1) were grown in a nursery, property of the Forestal Arauco Company (35° 18′ S, 72° 23′ W, 10 m a.s.l), located in the city of Constitución, central Chile. A mix of seeds from different mother trees represented each provenance. Seedlings were grown in 600 mL pots filled with composted bark of *P. radiata* which was combined with the slow release fertilizer BASACOTE 9 M, and cultured under ambient conditions of temperature and light. In both species, seeds were sown on March 2017 and maintained for 18 months under daily irrigation.

**Table 1:** Location and climatic parameters for the *Q*. *saponaria* and *C*. *alba* provenances under study.

Species	Provenance	Code	Latitude	Longitude	Altitude (m.a.s.l)	MAP	MAT	De Martonne aridity index
*Cryptocarya alba*	Hualañé	HU	34° 57ʹ	71° 46ʹ	145	837	13.5	35.6
	Los Queñes	QU	34° 59ʹ	70° 48ʹ	850	806	10.6	39.0
	Linares	LI	35° 56ʹ	71° 23ʹ	500	1092	12.8	47.7
	Coelemu	CO	36° 25ʹ	72° 40ʹ	50	1083	12.8	47.4
	El Carmen	CAR	36° 41ʹ	72° 21ʹ	120	1108	13.5	47.1
*Quillaja saponaria*	Vichuquén	VI	34° 38ʹ	71° 48ʹ	280	788	14.4	32.2
	Curepto	CU	35° 10ʹ	72° 04ʹ	420	839	11.6	38.7
	Pocillas	PO	35° 41ʹ	71° 52ʹ	200	851	13.6	36.0
	Cholguán	CHO	37° 09ʹ	72° 05ʹ	220	1376	12.7	60.5
	Cabrero	CAB	37° 20ʹ	72° 23ʹ	204	1206	13.2	51.8

*MAP* mean annual precipitation (mm), *MAT* mean annual temperature (°C).

The De Martonne aridity index was estimated as MAP/(MAT + 10).

### Experimental design

The field experiment was a randomized complete block design with seven blocks and included five provenances per species and a row-plot of 7-seedlings as the experimental unit (5 provenances × 7 blocks × 7 seedlings per block = 245 seedlings per species). No sun protection was provided for seedlings at the field test site. The site was fenced to avoid herbivore damage. Seedlings were hand planted during August 2018, in planting holes (25 × 25 × 30 cm) at a spacing of 1 × 1 m. All seedlings were watered once a month (4 L plant^−1^ month^−1^) during four months (December 2018 to March 2019) and no fertilizer was added to the planting holes.

### Survival and growth traits

Prior to planting the field experiments, all seedling were measured for height (Hi) and root collar diameter (Di) using a meter stick and a digital caliper, respectively. The slenderness index was estimated as the height to diameter ratio (Hi/Di). Then, one growing season after field establishment, seedlings were measured for H (Hp), D (Dp), and survival (SUR). SUR was measured as a categorical trait (i.e., alive seedling = 1, dead seedling = 0), and then expressed as percentage at plot level. Increments for D (INCd) and H (INCh) were calculated as the differences in those traits prior to planting with those one year after establishment.

### Gas-exchange measurements at the field experiment

During January 2019 light-saturated photosynthetic rate (A_sat_, *µ*mol CO_2_ m^−2^ s^−1^), stomatal conductance (g_s_, mol H_2_O m^−2^ s^−1^), and the derived intrinsic water use efficiency (WUE_i_ = A_sat_/g_s_) were measured in fully-developed leaves located on the upper third of the seedlings. These measurements were taken between 09:00 and 12:00 (local time) using an LI-6800 photosynthesis system (LI-COR Inc., Lincoln, NE, USA). The conditions of temperature, air CO_2_ concentration, and light source inside the chamber were set to 25 °C, 400 ppm, and 1800 mmol m^2^ s^1^, respectively.

### Data analyses

We conducted a first analysis to assess variations in survival, growth, and leaf-level physiology of species and provenances under study. Growth traits at the time of planting were analyzed with a lineal model considering provenance as a fixed factor, whereas survival, growth, and leaf-level physiology traits 1 year after outplanting were analyzed using a lineal mixed model including blocks as a random factor. Blocks were included to diminish the influence of environmental variation due to the heterogeneity of site conditions after the fire and thereby improve statistical power. The analyses of variance for each trait was carried out on a plot-mean basis (i.e., average of row-plot of 7-seedlings) for each species separately. To meet the assumptions of normality and constant variances, growth, and leaf-physiological traits were transformed according to the Box-Cox transformation when necessary^37^. In the case of survival, categorical values (i.e., 1, 0), were expressed in percentages at the plot level and transformed by the arcsine square root transformation. To correct those values that included 0 (zero), we added 1 (one) to each survival value. Mean comparisons were made using the Tukey test at P ≤ 0.05.

Because our interest was also to analyze the effect of seedling morphological attributes on outplanting performance, we used a simple regression model separately by species to assess (1) the relationship between seedling height and diameter at the time of planting with the survival one year after outplanting, and (2) the relationship between diameter, height, and survival with leaf-level physiological traits 1 year after outplanting. In both cases, we checked assumptions of normality and homogeneity of variances and the goodness of fit for the regression models was assessed by computing the coefficient of determination and the Pearson’s coefficient of correlation. In addition, we conducted a Path analysis with the aim to assess how the independent variables (Di, Hi, Dp, Hp, A_sat_ and g_s_), influenced the seedling outplanting survival. In this analysis we omitted the traits derived as ratios i.e., Hi/Di and WUEi (A_sat_/g_s_), to avoid multicollinearity.

Finally, a Principal Component Analysis (PCA) was carried out on all database with the aim to explore the relationship between seedling performance at the time of planting and after outplanting (Di, Hi, Dp, Hp, A_sat_, g_s_, and WUE_i_) with the climate at provenance locations where seed was collected using mean annual temperature and precipitation, and the De Martonne aridity index. Mean annual temperature and precipitation were taken from the Worldclim (www.worldclim.org) high-resolution dataset. All the statistical analyses were performed with SPSS version 18.0 software (SPSS Inc, Chicago, Illinois, USA) and INFOSTAT version 2018 (Group Infostat, Universidad Nacional de Córdoba, Argentina).

## Results

### Differences in height and diameter at time of planting

**Figure 2:**
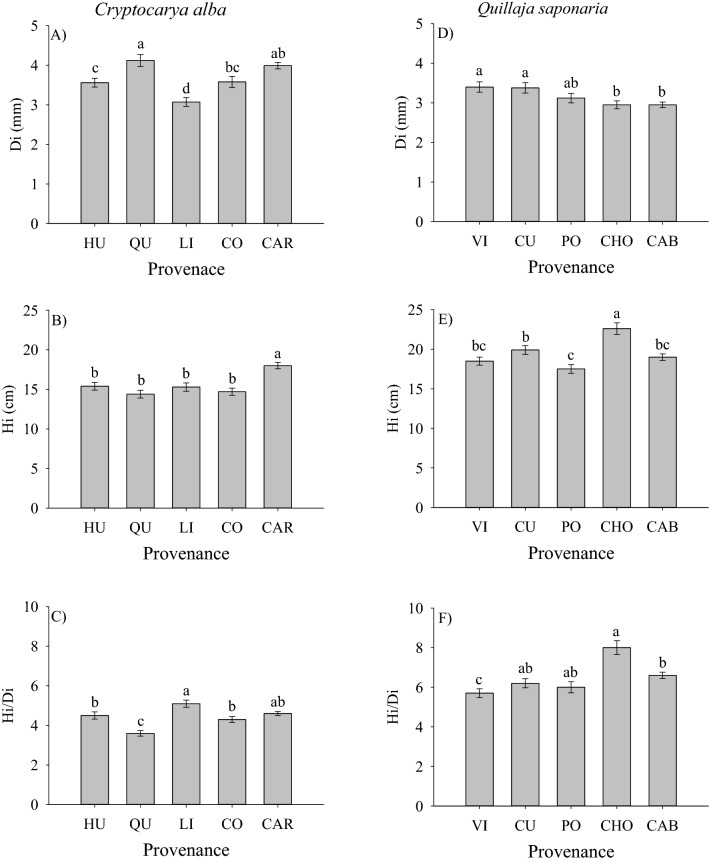
Seedling characteristics for *C*. *alba* and *Q*. *saponaria* at time of planting. *Di* root collar diameter, *Hi* height, *Hi/Di* height to diameter ratio. Different letters indicate significant differences among provenances (Tukey test; p ≤ 0.05). The error bars indicate the standard error. Codes for provenances are according to Table 1. Provenances are ordered from left to right by latitude of origin.

Both species exhibited differences in growth traits at the provenance level. In the case of *C*. *alba*, Di was higher in the provenances of Los Queñes (Pre Andean site), while Hi was higher in the provenance of El Carmen (interior site). The Linares provenance (Pre Andean site) had the lowest Di and exhibited the highest Hi/Di ratio (Fig. 2C). As Di was more variable among provenances (Fig. 2A) than Hi (Fig. 2B), the differences in Hi/Di are better explained by the differences in Di than in Hi. In the case of *Q*. *saponaria*, seedlings of the Cholguán and Pocillas provenances (both from interior sites) were the tallest and the shortest, respectively (Fig. 2E), while seedlings from Vichuquén and Curepto; from coastal sites, exhibited the thickest Di (Fig. 2D). As with Hi, the provenance Cholguán (from interior sites) had the highest Hi/Di ratio (Fig. 2F). The differences in Hi/Di for *Q*. *saponaria* were more associated to differences in Hi.

### Growth and survival in the field plantation and their relationship with seedling attributes at time of planting

**Figure 3:**
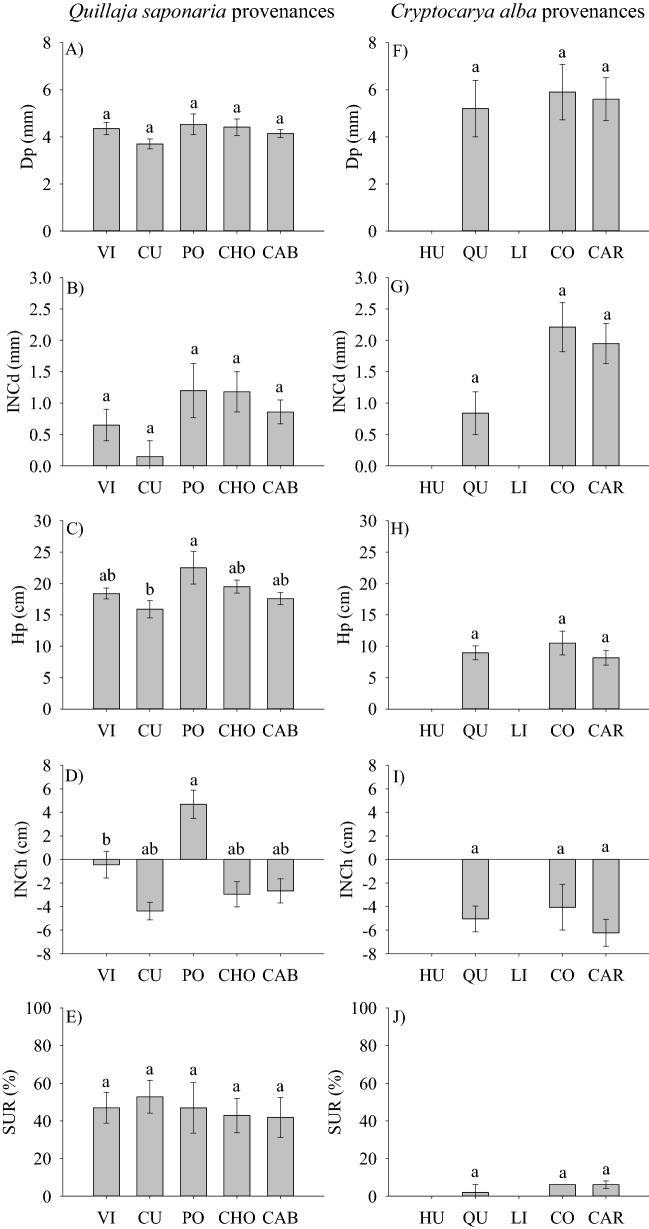
Survival (*SUR*), root collar diameter (*Dp*), height (*Hp*) and their respective increments in D (*INCd*) and H (*INCh*) for *Q*. *saponaria* and *C*. *alba* seedlings according to the provenance origin. Different letters indicate significant differences among provenances (Tukey test; p ≤ 0.05). The error bars indicate the standard error. Codes for provenances are according to Table 1. Provenances are ordered from left to right by latitude of origin.

**Figure 4:**
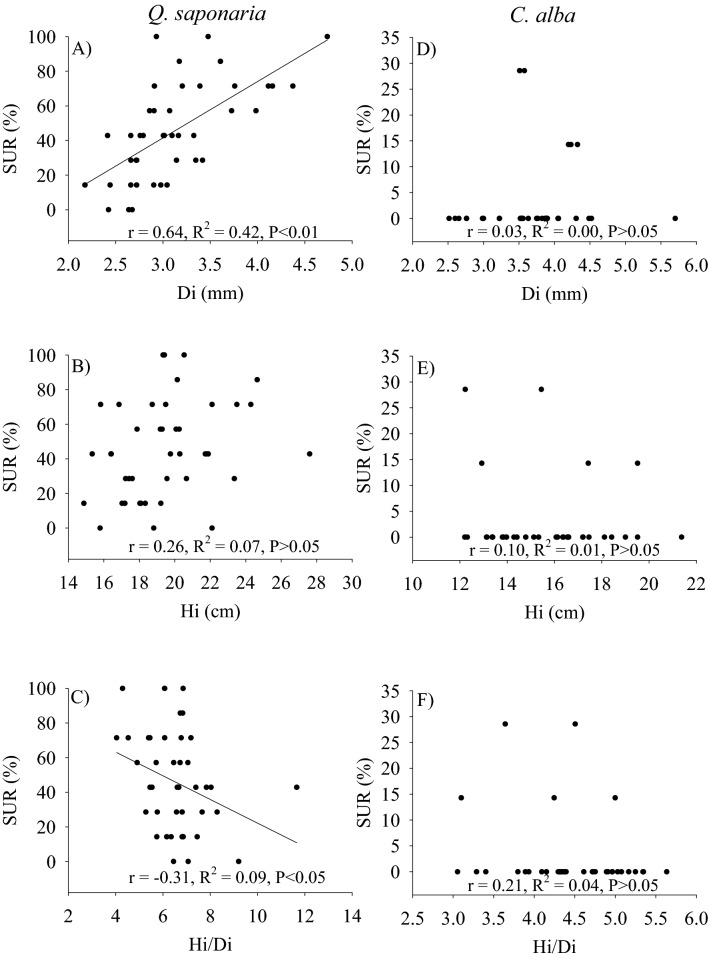
Relationship among the seedling attributes measured at the time of planting and survival measured 1 year after outplanting. Root collar diameter (*Di*), height (*Hi*), height to diameter ratio (*Hi*/*Di*), survival (*SUR*).

Provenances of *Q*. *saponaria* did not differ in survival and this trait ranged from 41 to 52% for provenances Cabrero and Curepto, respectively (Fig. 3E). Our results showed a significant provenance effects only for Hp and INCh (Table 2). Provenances Pocillas and Curepto exhibited the tallest and shortest seedlings, respectively (Fig. 3C), but INCh was higher and positive only in the Pocillas provenance. Most planting stocks of the other provenances exhibited negative height growth (Fig. 3D). Similar to survival, no provenance differentiation was found for INCd (Fig. 3B) and Dp ranged from 3.7 to 4.5 mm for provenances Curepto and Pocillas, respectively (Fig. 3A). In the case of *C*. *alba*, no differences in Dp, Hp, INCd, INCh and SUR were observed at the provenance level (Fig. 3F–I). SUR was very low in this species and ranged from 0 to 6% (Fig. 3J), while Hp and Dp of the surviving seedlings ranged from 8.1 to 10.5 cm, and from 5.2 to 5.9 mm, respectively. Most seedlings in all provenances exhibited negative INCh. The relationship between seedlings characteristics measured at the time of planting and SUR in the field showed low and poor correlations. Di and Hi/Di were positively and negatively correlated with SUR in *Q*. *saponaria*, but Hi exhibited no relationship with this trait (Fig. 4A–C). No significant correlations were found in *C*. *alba* (Fig. 4D–F).

**Table 2:** Analysis of variance for growth and survival traits of *Q*. *saponaria* and *C*. *alba* seedlings 1 year after outplanting.

Species	Traits				
	Dp	Hp	INCd	INCh	SUR
***Quillaja saponaria***					
P	0.209	**0.024**	0.365	**0.015**	0.893
***Cryptocarya alba***					
P	0.906	0.791	0.435	0.289	0.155

*P* provenance, *Dp* root collar diameter (mm), *Hp* height (cm), *INCd* increment in D (mm), *INCh* increment in H (cm), *SUR* survival (%).

Statistically significant values (*P* < 0.05) are in bold.

### Gas-exchange responses and its relationship with growth and survival

**Figure 5:**
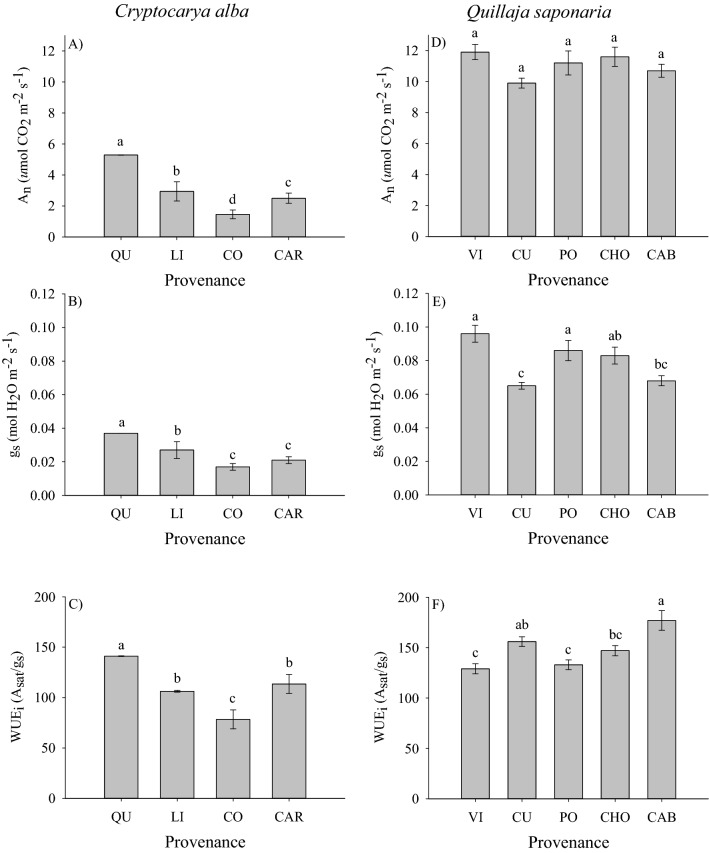
Gas exchange traits of *C*. *alba* and *Q*. *saponaria* seedlings according to the provenance. Different letters indicate significant differences among provenances (Tukey test; p ≤ 0.05). The error bars indicate the standard error. Codes for provenances are according to Table 1. Provenances are ordered from left to right by latitude of origin. In the case of *C*. *alba*, the provenance Hualañe (HU) had no alive seedlings after the post transplanting shock and this is why this provenance is missing from (A–C).

Provenances of *Q*. *saponaria* differed in g_s_ and WUE_i_ but not in A_sat_ (Fig. 5D, Table 3). The provenances of Vichuquén and Pocillas exhibited the highest g_s_ but the lowest WUE_i_. On the contrary, the provenance of Cabrero had the highest WUE_i_ while the provenance of Curepto exhibited low rates of g_s_ (Fig. 5E,F). In this species, SUR was positively correlated with A_sat_ (r = 0.54, *p* = 0.017) and with g_s_ (r = 0.58, *p* = 0.009). The relationship between other seedlings characteristics and physiological traits was non-significant. In the case of *C*. *alba*, the provenance Linares still had alive seedlings when gas exchange analysis was done (i.e., January 2019), but they died after the summer months. This is why this provenance appears with 0% survival, but with gas exchange measurements. Additionally, because no surviving seedlings were observed in the trial for the provenance of Hualañé, it was not possible to obtain physiological measurements. Therefore, the provenances of Los Queñes (pre-Andean provenance) and Coelemu (coastal provenance) consistently exhibited the highest and lowest values for all gas exchange traits under study (Fig. 5A–C). The relationship between seedlings characteristics and physiological traits of *C*. *alba* showed non-significant correlations (data not shown).

**Table 3:** Analysis of variance for gas exchange traits of *Q*. *saponaria* and *C*. *alba* seedlings 1 year after outplanting.

Species	Gas exchange traits		
	A_sat_	g_s_	WUE_i_
***Quillaja saponaria***			
P	0.063	** < 0.001**	** < 0.001**
***Cryptocarya alba***			
P	** < 0.001**	** < 0.001**	** < 0.001**

*P* provenance, *A*_*sat*_ light-saturated photosynthetic rates (*u*mol CO_2_ m^−2^ s^−1^), *g*_*s*_ stomatal conductance (mol H_2_O m^−2^ s^−1^), *WUE*_*i*_ Intrinsic water use efficiency (A_sat_/g_s_).

Statistically significant values (*P* < 0.05) are in bold.

### Path analysis of relationships between outplanting survival and independent variables

We used Path analysis to quantify simultaneously the direct and indirect contributions of seedling attributes and physiological responses on seedling outplanting survival (Table 4). The variable with the greatest direct effect on seedling SUR was g_s_, followed by Dp, A_sat_, Di, Hp, and Hi; but only Dp had directly significant negative effects on seedling survival (p < 0.05). In addition, this trait, via negatively affecting g_s_, indirectly affected seedling survival. Hp, A_sat_, and g_s_ had significant indirect effects on seedling survival through their interrelated effects. There was a significant indirect correlation between SUR with Hp and A_sat_ (correlation of 0.44 and 0.51) which is mainly determined by g_s_. Similarly, the significant indirect correlation between SUR and g_s_ was mainly determined by A_sat_.

**Table 4:** Path analysis of the relationships between outplanting survival and the various independent variables (growth and leaf-level physiology).

Factors	Direct effect	Indirect effect						Total correlation
		Di	Hi	Dp	Hp	A_sat_	g_s_	
Di	0.32	–	− 0.01	− 0.12	− 0.09	0.08	− 0.10	0.08ns
Hi	0.03	− 0.07	–	0.11	0.16	− 0.21	0.32	0.34ns
Dp	− 0.42**	0.09	− 0.01	–	− 0.05	0.12	− 0.21	− 0.48**
Hp	0.29	− 0.10	0.01	0.07	–	− 0.24	0.41	0.44**
A_sat_	− 0.33	− 0.07	0.02	0.15	0.21	–	0.54	0.51**
g_s_	0.57	− 0.06	0.01	0.16	0.21	− 0.31	–	0.58***

*Di* diameter at planting, *Hi* height at planting, *Dp* diameter after outplanting, *Hp* height after outplanting, *A*_*sat*_ photosynthesis after outplanting, *g*_*s*_ stomatal conductance after outplanting.

** and *** the correlation is significant at the p < 0.05 and p < 0.01 level.

### Principal component analysis of relationship among seedling performance and climate at provenance locations

**Figure 6:**
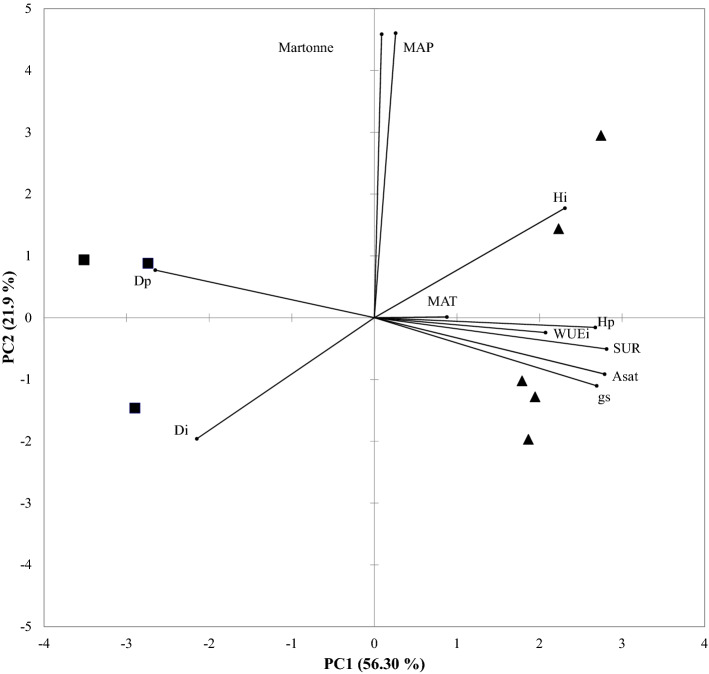
Principal Component Analysis among seedling performance and climate at provenance locations. Each squares and triangle represents a provenance of *C*. *alba* and *Q*. *saponaria*, respectively. In the case of *C*. *alba*, provenances HU and LI were absent due to they had no alive seedlings after the post transplanting shock. *SUR* survival after outplanting, *Di* diameter at planting, *Hi* height at planting, *Dp* diameter after outplanting, *Hp* height after outplanting, *A*_*sat*_ photosynthesis after outplanting, *g*_*s*_ stomatal conductance after outplanting, *WUEi* water use efficiency after outplanting, *MAP* mean annual precipitation, *MAT* mean annual temperature, *Martonne* The De Martonne aridity index.

The first (PC 1) and second (PC 2) principal components explained a significant proportion of the variability (78.2%) in the species and provenances under study (Fig. 6). The PC1 captures seedling performance, whereas the PC2 captures climate variables. SUR appeared to be positively correlated to the leaf-level physiological traits A_sat_ and g_s_, but negatively correlated to seedling diameter at the time of planting and after outplanting. Some provenances of *Q*. *saponaria* are associated to a higher SUR, A_sat_ and g_s_, whereas the provenances of *C*. *alba* are associated to larger diameters. Climatic variables did not show any correlation with the other variables, especially MAT.

## Discussion

The growth and survival were low and highly variable for both species. In the case of *Q*. *saponaria*, prior to planting seedlings differed in Di, Hi, and Hi/Di by provenance; however, with the exception of Hp no clear evidence of provenance differentiation was found for Dp and SUR 1 year after outplanting. The larger seedlings with the highest height increments were found in the provenance Pocillas, whose seed was collected at 24 km from the field test site and seedlings were the shortest prior to planting. As local genotypes performs better than distant genotypes^38^ it may be possible that this provenance have adaptations to the climatic conditions of the planting site that favored growth and positive height increments. Studies on sclerophyllous Mediterranean species have shown that survival improved using larger seedlings^19,21,23^, but in our study site characterized by low soil water availability and high temperatures during the growing season, seedling Hi was not related to field survival; however, the principal component analysis suggests that seedling Hp is associated to field survival. The Path analysis showed significant and negative direct effects of Dp on survival which; indirectly affecting g_s_, influenced survival. The initial stem diameter is a reliable indicator of field performance^5^ and our results suggests that, in sites with severe summer droughts, seedlings with ticker diameters could be associated to plants with higher transpiratory demands and stomatal conductances, with detrimental effects on seedling survival. It has been observed that small seedlings are better prepared to survive in dry environments^39^ because the low transpiring surfaces they develop and the consequent low risk of desiccation^4^. SUR at field was not correlated with Hi; which was corroborated by the Path analysis, but it was negatively correlated with Hi/Di, which was near the value of 6. Although the optimum value for Hi/Di that signifies good quality seedlings is unknown in *Q*. *saponaria*, it is recommendable to avoid the establishment of seedlings with excessive low or high Hi/Di values (i.e., stocky or spindly seedlings, respectively), as they have a low chance to survive in dry and windy sites^5^.

Both species experienced a high mortality, but this was particularly true for all provenances of *C*. *alba* that additionally experienced negative height growth. Negative height growth of *C*. *alba* was associated to a considerable decrease in leaf-level gas-exchange traits of seedlings submitted to a water restriction^40^. In addition, negative increments in height in *Quercus pagoda* Raf. and *Quercus phellos* L. were associated to the container type in which seedlings were cultured. Seedlings cultured in large containers were the largest in the nursery and experienced positive height growth at field^41^. Thus, the poor performance of provenances of *C*. *alba* in our study site might be explained by the shock experienced by small seedlings^15,42,43^. Planting small seedlings, as those of *C*. *alba* in our experiment (i.e., Hi < 20 cm), implies a reduced volume exploration by roots and consequently an insufficient water uptake and survival^22^. *C*. *alba* seedlings were also stocky prior to planting (Hi/Di in the 3–4 range), which might have contributed to low field survival^5^. However, in our study, there was not enough evidence supporting the effect of the seedling size prior to planting on the outplanting growth and survival of the different provenances. In this respect, in the sclerophyllous species *Q. ilex* and *Quercus coccifera* L., no significant correlations were found between field survival and seedling traits at nursery^14,44,45^. It seems that in harsh environments; as our study site that was additionally severely damaged by fire, it is not clear which seedling attributes determines establishment success^46^. The estimated burn severity index (i.e., RdNBR) indicated a high severity damage by fire in the study area. This condition probably changed the spatial patterns of the soil properties, exacerbating microsite variation and negatively affecting outplanting performance of both species, but this needs to be further investigated.

Unlike growth and survival, our study showed that *Q*. *saponaria* and *C*. *alba* exhibited differences among provenances in gas exchange parameters. The Principal component analysis showed no association between leaf-level physiological traits with climate variables but it reveals that most of the provenances *Q*. *saponaria* are associated to a higher SUR, A_sat_ and g_s_. The analysis of variance indicated that, with the exception of A_sat_, provenances of this species differed in g_s_, and WUE_i_. We found low values for g_s_ and higher WUE_i_ in the provenance Cabrero, a provenance originating from sites with mean annual precipitation of 1206 mm year^−1^, sandy soils with low available water capacity, and an extended dry season from 5 to 7 months^47^. The superior WUE_i_ in the Cabrero provenance may be attributed to a reduction in g_s_ because A_sat_ was not different among provenances. The likely natural adaptation of that provenance to the drier conditions from which it was originated might have allowed it a higher stomatal control and water conservancy. On the contrary, the Vichuquén provenance maintained superior levels of g_s_ but had low WUE_i_. As this provenance is originated from sites close to the coast (20 km from the coast) with higher humidity and metamorphic soils, when established in the dry site of the study area, it became less water use efficient as an apparent adaptation to its local growing conditions. In a similar experiment it was found a higher WUE_i_ and low g_s_ in a provenance of *Q*. *saponaria* originated in sites with a Mediterranean-type climate and whose seed was collected at a similar latitude than the Cabrero provenance of our experiment^48^. Similarly, the authors found superior levels of g_s_ and low WUE_i_ in a provenance whose seed was collected in a site located at 10 km from the coast. In *Q*. *saponaria*, the relationship between SUR with A_sat_ and g_s_ was positive but needs to be interpreted with caution. A_sat_ was not different among provenances and the provenances that exhibited higher g_s_ did not exhibit a clear superiority in survival._._ Our path analysis showed complex relationships among A_sat_ and g_s_ in indirectly affecting seedling survival. As both traits are closely related, our interpretation is that the control of stomatal closure in the summer months after the transplanting shock will promote a high survival in the harsh conditions of the planting site, corroborating the importance of stomatal regulation as a mechanism to prevent water loss and assure survival in Mediterranean sclerophyllus species^12,13^. In the case of *C*. *alba*, the contrasting differences in gas exchange between the provenances Coelemu and Los Queñes might be also related to its adaptation to specific site conditions. The provenance of Los Queñes comes from a pre-Andean site located at an altitude of 850 m.a.s.l. (Table 1); characterized by low mean annual temperature and negative temperatures in winter, and seedlings were able to sustain the highest A_sat_ and WUEi of the provenances under study. This provenance might have evolved to optimize water use under cold condition as surviving under these conditions requires great stress tolerance, phenotypic plasticity or both. On the contrary, the provenance of Coelemu is a coastal provenance from altitudes close to the sea level and sites with warmer temperatures, and seedlings of this provenance were among the lowest performers in leaf-level physiology. The behavior of this provenance suggests that it follows the specialization theory^49^ in which genotypes adapted to favorable conditions; a coastal environment in this case, may have a decreased performance in restrictive environments, represented by the harsh conditions of our planting site. This could imply a low adaptability of coastal provenances when transferred to more dry sites, which needs to be further investigated.
